# Neuroprotective Potential of Cell-Based Therapies in ALS: From Bench to Bedside

**DOI:** 10.3389/fnins.2017.00591

**Published:** 2017-10-24

**Authors:** Serhiy Forostyak, Eva Sykova

**Affiliations:** ^1^Centre of Reconstructive Neuroscience, Institute of Experimental Medicine (ASCR), Czech Academy of Sciences, Prague, Czechia; ^2^Department of Neuroscience, 2nd Faculty of Medicine, Charles University, Prague, Czechia; ^3^Institute of Neuroimmunology, Slovak Academy of Sciences, Bratislava, Slovakia

**Keywords:** stem cells, neurodegeneration, neuroprotection, clinical trials

## Abstract

Motor neurons (MN) degeneration is a main feature of amyotrophic lateral sclerosis (ALS), a neurological disorder with a progressive course. The diagnosis of ALS is essentially a clinical one. Most common symptoms include a gradual neurological deterioration that reflect the impairment and subsequent loss of muscle functions. Up-to-date ALS has no therapy that would prevent or cure a disease. Modern therapeutic strategies comprise of neuroprotective treatment focused on antiglutamatergic, antioxidant, antiapoptotic, and anti-inflammatory molecules. Stem cells application and gene therapy has provided researchers with a powerful tool for discovery of new mechanisms and therapeutic agents, as well as opened new perspectives for patients and family members. Here, we review latest progress made in basic, translational and clinical stem cell research related to the ALS. We overviewed results of preclinical and clinical studies employing cell-based therapy to treat neurodegenerative disorders. A special focus has been made on the neuroprotective properties of adult mesenchymal stromal cells (MSC) application into ALS patients. Finally, we overviewed latest progress in the field of embryonic and induced pluripotent stem cells used for the modeling and application during neurodegeneration in general and in ALS in particular.

## History, genetics, and clinics of ALS

In 1848, Aran described for the first time a malady later become known as an amyotrophic lateral sclerosis (ALS). He reported 11 cases of the disease featuring a focal wasting and paresis, weakness and cramps in the upper extremities, and fatal end within 2 years (Aran, 1848). Aran proposed that the disease had been inherited from the parents. In 1873, Jean-Marie Charcot reported that ALS was never inherited, and that was the main reason for delineating ALS from muscular atrophy (Charcot, 1881). The view that ALS is rarely connected with family history persisted for almost 100 years. A new era in the field has started when several genes were linked with familial (FALS) and sporadic ALS (SALS) cases. It is thought that around 5–10 percent of all ALS incidents have a family history, whereas the rest are sporadic (Bento-Abreu et al., 2010; van Es et al., 2010; Andersen and Al-Chalabi, 2011). The mutations in the following genes have been found to result in FALS: superoxide dismutase 1 (*SOD1*), *TARDBP, Ubiquilin 2, Alsin, Senataxin, FUS, Angiogenin, SIGMAR1* (Rosen et al., 1993; Hadano et al., 2001; Hand et al., 2002; Sapp et al., 2003; Chen et al., 2004; Nishimura et al., 2004; Gitcho et al., 2008; Kabashi et al., 2008; Sreedharan et al., 2008; Vance et al., 2009; Elden et al., 2010). ALS has been recently associated with frontotemporal dementia, (FTD, ALS/FTD). A GGGGCC hexanucleotide repeat in the intron of protein C9ORF72 has been demonstrated to cause an alternative splicing of this protein that is leading to similar pathological events in two diseases (DeJesus-Hernandez et al., 2011; Renton et al., 2011). Another pathological features of ALS and FTD are TDP-43 and p62 positive cytoplasmic depositions in the hippocampus and cerebellum (Achi and Rudnicki, 2012; Mahoney et al., 2012). The main differences between ALS/FTD patients and classical FTD cases are presences of psychiatric signs and the distribution of TDP-43 inclusions: SALS primarily features TDP-43 pathology in the spinal cord, patients with FTD primarily affect the cortex, while FTD-ALS patients have TDP-43 pathology in both areas (Geser et al., 2009; Neumann et al., 2009). The genetic screening of US population demonstrated that TDP-43 expansion occurs in 12% of familial FTD and 22.5% of FALS (DeJesus-Hernandez et al., 2011), while European population screening showed higher prevalence in FALS (46%), followed by familial FTD (29%) and SALS (21%) (Renton et al., 2011).

Despite diverse genetic backgrounds, SALS and FALS are clinically indistinguishable, 95% of all ALS cases are sporadic, and the other five percent have a genetic background. The clinical hallmark of both types of ALS is a progressive deterioration of neurological functions correlated (clinically and pathologically) with loss of primary and secondary MN, sparing of the oculomotor and the spinal Onuf's nuclei, coexistent neurogenic atrophy, weakness, and fasciculations caused by secondary MN degeneration, together with hyperactive deep tendon reflexes, pyramidal tract signs, and increased muscle tone (Borasio and Appel, 2003). Patients present a wide range of diverse clinical outcomes regarding disease onset, rate of progression and survival (Burkhardt et al., 2013). Disease's symptoms are typically asymmetrical. Some 20–30% of all cases have bulbar onset, with more than 50% of bulbar symptoms in older women. In FALS minor pathological changes could be diagnosed in the spinocerebellar tracts, typically without accompanying symptoms. Most commonly, the disease strikes people between the ages of 40 and 70, although the early onset is not exceptional. Unlike other neurodegenerative maladies, ALS is not age-related disease. However, aging is one of a many risk factors. Incidence of ALS is fairly uniform 1–2 per 100,000 individuals, except for an elevated incidence in Kii peninsula of Honshu island and Guam (Kuzuhara and Kokubo, 2005; Steele, 2005). A lifetime risk of ALS development approaches 1/400-1/700 with a somewhat more frequent occurrence in males than in females (ratio is ≈ 1.5; Johnston et al., 2006; Bento-Abreu et al., 2010).

## Current treatment and management of ALS

Considering a great diversity of genetic and clinical forms of ALS, every therapeutic attempt could be regarded as experimental. Nevertheless, as for now a standard therapy for ALS includes an antiglutamatergic agent Riluzole that, however, does not alter the natural history of the disease (Lacomblez et al., 1996). Riluzole (100 mg**/**day), which reduces the presynaptic release of glutamate, remains the only effective drug that slows disease progression and extends the patients lifespan by 2–3 months (Lacomblez et al., 1996; Sykova et al., 2017). Additionally, all patients receive palliative or symptomatic therapy such as a non-invasive positive pressure ventilation (Hardiman, 2011), prescription of anticholinergic drugs (such as trihexyphenidyl, amitriptyline, or atropine) or the use of a portable suction machine if drooling is troublesome. Baclofen or diazepam might be used to deal with spasticity (Mustfa et al., 2006). Dysphagia could be managed by modifying food and fluid consistencies, postural advice, and in extreme cases of bulbar involvement, by gastrostomy or cricopharyngomyotomy. The results of such therapy are unsatisfactory, current clinical management is still extremely limited and novel therapeutic approaches are in an active search. The presymptomatic or at least the early diagnosis of ALS could offer wider possibilities for prevention and for the treatment of this devastating disease. Therefore, in the future screening of patients with FALS for mutations in the SOD1, TARDBP, FUS, and a several other genes might offer benefits in the diagnosis and treatment of ALS. Sadly, over 60 per cent of ALS patients die within 2–5 years of presentation mostly from pulmonary insufficiency with infections, with < 10 per cent survive rate longer than 8 years (Kiernan et al., 2018). Considering that the above therapies just improve patient's quality of life, but do not extend his/her survival the main task of treatment in the terminal stages is to keep patients as comfortable as possible (McGeer and McGeer, 2005).

## Neuroprotective strategies in ALS

Considering that the disease affects MN at different levels of the central nervous system (CNS), a neuroprotective strategy should aim to restore affected tissue homeostasis throughout the entire nervous system. Numerous attempts have focused on antiglutamatergic, antioxidant, antiapoptotic, anti-inflammatory, and neurotrophic molecules, as well as on gene therapy and stem cell application. These and other molecules are able to reach the MN after systemic (intravenous, intraarterial), local (intrathecal, intraspinal, intracerebral, etc.) or combined application, as it has been shown that the blood brain barrier (BBB) in ALS is also compromised (Garbuzova-Davis et al., 2007).

### Antiglutamatergic therapy

Antiglutamatergic Therapy has currently shown the best results in clinical trials. As already mentioned, the only anti-ALS medicine approved for the treatment of patients is Riluzole. The kynurenine pathway (KP), a major route for the metabolism of tryptophan, has been shown to play role in ALS. The KP excitotoxic catabolites such as N-methyl-D-aspartate receptor agonist quinolinic acid and the neuroprotective NMDA receptor antagonist kynurenic acid are involved into crosstalk between the CNS and immune systems, by modulating cell proliferation. Few companies such as Sanofi-Aventis or Teva Neuroscience developed KP inhibitors (Teriflunomide and Laquinimod, respectively), have entered clinical trials (Chen et al., 2009). Memantine, novel anti-excitatory drug is a non-competitive excitotoxic N-methyl-D-aspartate (NMDA)-receptor antagonist, has been shown to delay the loss of hind limb motor activity and extend the survival of SOD1^G93A^ mice (Wang and Zhang, 2005).

### Antioxidant therapy

Antioxidant Therapy aimed at ameliorating oxidative stress, could provide a possible healing effect in ALS patients. However, clinical trials examining the application of vitamin E, acetylcysteine, methylcobalamine, glutathione, or coenzyme Q10 (CoQ10) indicate that these drugs are ineffective in ALS patients (Levy et al., 2006; Kaufmann et al., 2009). An antioxidant peptide called SS-31 has been shown to improve mitochondrial dynamics, resulting in a significant extension of survival, better motor activity, decreased MN loss and reduced immunostaining for oxidative stress markers in G93A mice (Petri et al., 2006).

### Immunotherapeutic strategies

Immunotherapeutic Strategies to combat ALS also could be an attractive therapeutic approach. Active vaccination with misfolded mSOD1 in the G37R SOD1 mouse model of FALS has been tried, resulting in the reduced loss of spinal cord neurons and a modest but statistically significant increase in life expectancy (Urushitani et al., 2007; Brody and Holtzman, 2008). However, much work remains to be done before clinical trials could be started.

The discovery of neurotrophic factors (NTF), their anti-apoptotic effect and the ability to promote the MN survival during development, made these molecules attractive candidates for the treatment of neurodegenerative disorders affecting motor system (Appel, 1981; Gould and Oppenheim, 2011). Increasing number of studies implicate an impaired production of vascular endothelial growth factor (VEGF) by MN, rather than a lack of functional receptors, is associated with ALS. The exogenous VEGF has been shown to cause a direct neuroprotective effect via the expression of VEGF-receptors in MN (Van Den Bosch et al., 2004). Various routes of insulin-like growth factor-1 (IGF-1) or VEGF delivery, was reported to slow disease progression and improved lifespan in animals studies (Kaspar et al., 2003; Nagano et al., 2005; Wang et al., 2007). Interestingly, clinical trials utilizing the subcutaneous delivery of IGF-1 failed to show a beneficial effect, mainly due to a reduced bioavailability of IGF-1 (Sorenson et al., 2008; Howe et al., 2009). The combined usage of IGF with IGF-binding protein 3 (IGFBP), also called IPLEX, significantly increased the serum half-life time of IGF-1 and proceeded toward an early-phase clinical trial. Despite a great deal of debate surrounding the effectiveness of IPLEX (Bedlack et al., 2009; Gould and Oppenheim, 2011), clinical trial employing a delivery of VEGF protein into ALS patients CSF is currently in progress (http://clinicaltrials.gov, identifier # NCT01384162 and NCT008005501).

One should also consider that MN exhibit trophic heterogeneity, that is they respond to a distinct type/s of NTFs during their development (Kanning et al., 2010). Studies on glial cell line-derived neurotrophic factor (GDNF) knockout mice showed a dramatic and restricted loss of small ventral lumbar myelinated axons (γ-MN) and spared large myelinated axons (α-MN) (Gould et al., 2008). Hence, a monotherapeutic strategy might bring some improvements in motor activity or even extend survival, but this demands distinct NTFs that will target specific types of MN. This concern might be resolved by using cocktails of NTFs delivered in such a way so that they would be able to pass through the BBB in an efficient and controlled way. Alternatively, application of stem cells, which are well-known to have paracrine properties, could serve as a vehicle for the delivery of NTFs. Stem cells research has reached a stage when unlimited number of non-engineered and engineered neuroglia could be produced and used for therapeutic purposes aiming at protection, repair, or replacement of affected cells in disorders affecting the brain and spinal cord, thus bringing a new hope for patients.

## Cell-based therapies

A new avenue has been opened for basic research and regenerative medicine with the discovery of stem cells (SC) and their regenerative capacities. By using a SC terminology one would expect to deal with a cell with a pluri- or multipotent features and unlimited self-renewal capacities. Pluripotency means that the cell is capable to differentiate into any mature cell type in the organism originating from all three germ layers (Nistor et al., 2005; Lee et al., 2007). SC classification considers the tissue of origin, the developmental stage when cells appear and could be isolated in the organism. Current research is dealing with the following types of SC: (1) embryonic (ESC), a pluripotent cells that could give rise to any cell in the body; (2) fetal (FSC), a multipotent cells that could give rise to any cell of certain germ layer; (3) somatic (adult-derived), multi- or oligopotent cells found in different tissues of the fully developed organisms; and 4) induced pluripotent (iPS), similar to ESC, but generated from mature somatic cells after the artificial introduction of transcriptional factors (Takahashi and Yamanaka, 2006).

### Mesenchymal stromal cells (MSC)

MSCs are attractive and accepted target for use in cell-based therapies (autologous application). MSC are oligopotent cells that could be isolated by a relatively simple procedure (Forostyak et al., 2016a) from bone marrow (BMSC), umbilical cord (UMSC), fat tissue (AMSC), Wharton's jelly and other fetal tissues. To meet the “mesenchymness” criteria every cell should meet following criteria: ability for extensive *in vitro* growth on plastic; differentiate *in vitro* into chondrocytes, osteocytes and adipocytes; express “mesenchymal” surface markers (Mezey et al., 2000; Krause, 2002; Dominici et al., 2006; Forostyak et al., 2016a). It is interesting to note that despite great similarities in features and properties, MSCs of different origin display certain differences in growth rate, surface markers expression and even physiological activities (Forostyak et al., 2016b). Several groups also reported MSC differentiation toward functional neural phenotype (Tropel et al., 2006).

A therapeutic potential of MSC is very broad and still hides many unknown features to be exlored. However, it is generally accepted that despite the MSC's origin, they primarily act via the growth factors (GF) secreted either into the growth medium or within the recipient's tissue (paracrine function). A GF misbalance in the organism triggers cascades of intracellular changes that may result into various pathological states. Therefore, MSC ability to secrete a cocktail of growth factors brought attention for their use in regenerative medicine in general, and is very promising to use for the purpose of neuroprotection and neuroregeneration. We earlier reviewed available information about growth factors and corresponding genes that have been reported to be secreted by MSC (Forostyak et al., 2013b). Here, we will just name GFs that are known to contribute to ALS and were successfully tested *in vivo* for the purpose of neuroprotection or disease modification, those are: glia cell-line derived neurotrophic factor (GDNF), insulin growth factor type-1 (IGF-1), brain-derived neurotrophic factor (BDNF), neural growth factor (NGF), VEGF and others that play less significant role in ALS pathology. Apart from a paracrine effect, we have recently reported that after an intrathecal or combined application of MSC a level of apoptosis and inflammation decreases leading a better survival of host MN in host tissue (Forostyak et al., 2014). Moreover, MSC has been shown to repair a defective extracellular matrix (ECM) structure called perineuronal net (PNN) surrounding these MN in SOD1 rat transgenic model of ALS, by up-regulation of some chondroitin sulfate proteoglycan levels (CSPG) typically up-regulated at the end of a critical period of PNN development (Carulli et al., 2010; Forostyak et al., 2014). We could speculate that this finding could be indicating a reactivation of adult neural plasticity within a recipient's organism after MSC application. Notably, MSC do not stimulate alloreactivity, are able to pass a MHC barrier, and thus enabling appliocation between HLA-mismatched individuals (Le Blanc, 2003; Aggarwal and Pittenger, 2005; Rice and Scolding, 2008).

The above properties make these cells a very attractive target for neuroprotective and neuroreparative therapy. Increasing number of reports has led to the preclinical trials. These studies demonstrated positive effects of MSC on motor activity and survival after being delivered via various routes (mostly intrathecal, but also intravenous, intraspinal, intramuscular, or combined) using rodents' models of ALS (Mazzini et al., 2004; Garbuzova-Davis et al., 2008; Kim et al., 2010). Our preclinical study employing intrathecal (cisterna magna) injection of human MSC into symptomatic SOD1^G93A^ transgenic rats, demonstrated prolonged survival of MSC-treated animals by more than 2 weeks compared with the vehicle-treated group, and also demonstrated decreased markers of inflammation in host CSF (Forostyak et al., 2014). Another studies using a combined (intraspinal and intravenous) BMSC transplantation, a part of an increased motor activity and survival and attenuated proliferation of microglial cells (Boucherie et al., 2009; Forostyak et al., 2011). Interestingly, we have noticed that despite a broad application of MSC in animal studies and in clinical trials involving patients, little has been known about MSC's physiology. The stem cells physiology may enable a better control over the graft and even further improvement of regenerative potential of cells prepared for human application. This knowledge may change and further improve effect of cell-based therapies of neurodegenerative and other types of diseases. These questions have been raised in several of our studies, where we have studied stem cells physiology with a help of Ca^2+^ signaling, ICC, electrophysiological methods and other methods of molecular biology. Ca^2+^ signals play a crucial role in the differentiation, proliferation and survival of stem cells starting from the early stages and later on mature cells (Forostyak et al., 2013a, 2016b). Dysregulation of calcium ([Ca^2+^]_i_) homeostasis is also crucial in the pathophysiology of ALS, and impaired [Ca^2+^]_i_ in the cytoplasm of neurons is a potential mechanism of decreased cell survival (Dafinca et al., 2016). MSCs effects are dose- and passage-dependent, and we showed that MSC from earlier passages (up to the fifth) are more suitable therapeutic application due to their stability, anti-inflammatory and neuroprotective effects (Choi et al., 2010; Forostyak et al., 2016a). Similar outcomes were achieved by the administration of 10^6^ cells in asymptomatic SOD1 animals, while 10^5^ cells failed to change the prognosis of the disease (Habisch et al., 2007; Kim et al., 2010).

### Embryonic and induced pluripotent stem cells

The advances in stem cell research demonstrated a great potential of cell-based approach in the treatment of currently incurable diseases and this brings hope for patients and their families, especially in the case of neurodegenerative disorders or neurotrauma. The Nobel Prize in Physiology or Medicine for “The discovery that mature cells can be reprogrammed to become pluripotent” highlights the importance of SC research in general and particularly iPS technology (Takahashi and Yamanaka, 2006).

At the moment several groups are working on substitution of defective neurons with donor stem cell-derived neuronal progenitors. The idea behind is that the grafted cell will integrate after transplantation into existing neural circuits and take over the functions of defective cells (Kallur et al., 2006; Lindvall and Kokaia, 2006). It has been reported that MN generated from ESC are not just able to maintain typical motor neuronal phenotype *in vitro*, but also to functionally incorporate into host CNS (Wichterle et al., 2002; Papadeas and Maragakis, 2009). A glial restricted precursors (GRP) or human neural stem/progenitor cells (hNSC) seem to have a greatest potential in ALS research. These cell types were described to modify ALS prognosis, to reduce MN death and to establish functional synapses with structural integration into the motor circuitry (Lepore et al., 2008; Xu et al., 2009). In ALS functional integration of grafted cells is crucial. However, MN replacement would also expect an axonal outgrowth and neuromuscular junction (NMJ) formation to innervate muscle fibers that otherwise are dedicated to be atrophied. We found only few studies that have demonstrated formation of new functional connections between grafted SC and the recipients' muscles after the grafting (Deshpande et al., 2006; Yohn et al., 2008; Gowing and Svendsen, 2011).

Taking into the consideration above limitations, generation and grafting of protective cells that will support the remaining MN might be more effective. It is known that in ALS astrocytic reactivity with proinflammatory transcriptional and translational profiles exacerbates motor neurons (MNs) dysfunction (Sun et al., 2015). These specific pathological events could be targeted by the application of either ES or iPS cells that were directed either toward progenitor or mature astrocytic phenotypes prior to transplantation (Popescu et al., 2013; Kondo et al., 2014).

The possibility of hyperproliferation and the formation of teratomas is always a risk related with ESC-/iPSC-derived neural progenitors (Seminatore et al., 2010). Latest preclinical studies demonstrate that this issue could be controlled, thus Food and Drug Administration (FDA) has permitted a phase I clinical study testing the safety of the intraspinal grafting of neural stem cells, into patients with confirmed ALS (http://clinicaltrials.gov, identifier #NCT01348451) (Gowing and Svendsen, 2011; Lunn et al., 2011). A transplantation of glial precursor cells or mature astroglia is another quit realistic approach to promote neuroprotection and trophic effects, that will support the failing motor neurons (Robberecht and Philips, 2013). These cells generated from somatic tissues, even from elderly patients, could be further reprogrammed to reach a pluripotent stage, and later differentiated toward neural/astroglial morphology of interest ready for delivery into the patients (Dimos et al., 2008; Hall et al., 2017). This alternative to embryonic/fetal cells, if isolated from affected individuals, could be used either as an *in vitro* model of neurodegenerative diseases, helping us to understand the mechanisms underlying the pathological processes, or could be used as therapeutic agents after avoiding the risk of graft rejection or the opposite, graft-vs.-host-disease if the donor and recipient is a same individual. The cutting-edge of modern cell biology is the generation of functional induced motor neurons or astrocytes from the patient's own fibroblasts, which after transplantation could protect the dying MN (Son et al., 2011; Hall et al., 2017).

Considering that neurodegenerative diseases often develop in elderly patients, there are some concerns related to the “quality” of stem cells if generated from an aged and sick organism and whether these cells might be used for cell replacement, neuroprotection and neuroregeneration. So far reports addressing this question are quit controversial. Dimos et al. by using the example of iPS cells generated from an 82-year-old woman with a FALS, showed that these cells possess the same properties as do ESC and that they could differentiate into MN (Dimos et al., 2008). However, in the case of iPS cells, which are generated using a cocktail of overexpressed transcriptional factors transferred to skin fibroblasts (or other somatic cells) by transfection of viral vector infection, it is necessary to note that we do not yet know whether the human use of these cells will not increase a risk of genetic modification of both donor and recipient cells (Wichterle and Przedborski, 2010). Concerns are also about the route of transplantation and the developmental stage of the pluripotent cells. It is necessary to keep in mind that the manipulation with the cells that are fully differentiated to the neural or neuronal phenotype is extremely difficult. Therefore, the cells at the precursor stage are potentially better candidates for the transplantation. According to the publicly available information, several clinical trials that are engaging human neural stem cells application to evaluate the safety and efficiency of such a therapy of ALS are currently recruiting patients or are on-going (Glass et al., 2012).

### Clinical trials of stem cell therapy in ALS

*In vivo* experiments using rodent models of FALS formed a platform for clinical trials involving patients (Vercelli et al., 2008). We have analyzed supported past, current and future clinical trials from the clinicaltrial.gov website and summarized them into the overview table (Table 1). It is obvious that the number of phase 1/2 clinical trials is increasing annually. Mostly, these are safety studies involving small amounts of patients. Unfortunately, great majority of the trials does not describe details about the dosage, type of the cells, criteria for patients monitoring and does not report a feedback about the study outcomes. This complicates interpretation of the data and creates obstacles for further clinical application of various cell types. The majority of approved/available clinical trials employ mesenchymal stromal cells (MSCs) of different origin (mostly bone marrow) for the therapy of ALS. It could be explained mainly by the easiness of derivation and manipulation with autologous cells from patients, legal issues and long history of clinical application of bone marrow derived cells. On the other hand, this cells are quite unique, especially for their paracrine properties for detailed overview see (Forostyak et al., 2013b).

**Table 1:** An overview of clinical trials involving cell-based therapy to treat ALS (modified from www.clinicaltrials.gov).

**Cell type (additional intervention)/dose**	**Stage of disease/place of cell delivery**	**Evaluation time after Tx**	**Country/Company/Identifier (NCT and other)**	**Phase of the trial/stage/estimated trial end**	**Results**	**Side effects**	**PI/References**
**1**. Intrathecal autologous bone marrow mononuclear cell transplantation/na	Na/intrathecal	2 years	India/Neurogen Brain and Spine Institute/NCT02242071	Phase 1/Completed/September 2016	na	na	Neurogen Brain and Spine Institute/na
**2**. Autologous intrathecal administration of hematopoietic stem cells/na	Na/intrathecal	During the procedure and at 1st, 2nd, 3rd, 6th, and 12th month after the procedure	Mexico/Hospital Universitario Dr. Jose E. Gonzalez/NCT01933321; NGBSI-10	Phase 2, phase 3/Completed/April 1, 2015	na	na	David Gomez Almaguer/na
**3**. Autologous bone marrow mononuclear cell transplantation on the survival duration	Na/intrathecal and intramuscular routes	Retrospective Control Study	India/Neurogen Brain and Spine Institute/NCT01984814; NGBSI-04	Phase 2/Completed/November 2013	na	na	Alok K Sharma/na
**4**. Mesenchymal stem cells/1 × 10^8^ mesenchymal stem cells +10cc normal saline	Na/intraspinal injection of	Before transplatation and at 6 months, 12months, 18 months, and 24 months after transplantation	Iran/Isfahan neurosciences research center/NCT02116634;rokhsareh	Phase 1, 2/withdrawn/January 2016	**Withdrawn prior to enrolment**	na	Dr. Keivan Basiri /na
**5**. Human glial restricted progenitor cells (hGRPs; Q-Cells®)/na	Na/5-10 intraspinal grafts: **Cohort 1**, Unilateral lumbar surgical transplantation of Q-Cells dose level 1; **Cohort 2**, Unilateral cervical surgical transplantation of Q-**Cells** dose level 1; **Cohort 3**, Unilateral cervical surgical transplantation of Q-**Cells** dose level 2; **Cohort 4**, Unilateral cervical surgical transplantation of Q-Cells dose level 3; **Cohort 5**, Unilateral cervical surgical transplantation of Q-Cells dose level 4; **Cohort 6**, Unilateral cervical surgical transplantation of Q-Cells dose level 5	Before transplantation and during 9 months after Tx (Parallel Assignment). Following the 9-month study period, subjects who consent will continue to be followed for safety and efficacy long-term in a separate protocol.	/Q Therapeutics, Inc./NCT02478450; QALS-101	Phase 1/2a/not yet recruiting/April 2020/	na	na	Q Therapeutics, Inc./na
**6**. Autologous mesenchymal stem cells/ **Group 1**: single intrathecal dose of 1 × 10^7^cells; **Group 2:** single intrathecal dose of 5 × 10^7^ cells; **Group 3:** one intrathecal dose of 5 × 10^7^ cells followed one month later by a second intrathecal dose of 5 × 10^7^ cells; **Group 4:** single intrathecal dose of 1 × 10^8^ cells; **Group 5:** one intrathecal dose of 1 × 10^8^ cells followed one month later by a second intrathecal dose of 1 × 10^8^ cells	Five treatment groups of up to five patients each: **Groups 1, 2**, and **4** will receive a single dose of cells. **Groups 3** and **5** will receive 2 doses of cells separated by one month. Intrathecal injections into new subjects will be timed so that there is a minimum of 1 week between subject injections	Initial clinical follow-up will be weekly with scheduled blood, CSF and magnetic resonance imaging (MRI) evaluations. Regular assessment until death or for a minimum of 2 years after the final infusion. After 1 month, patients will have clinical evaluations at 3 month intervals, or earlier if indicated by clinical status.	US/Mayo Clinic/NCT01609283; 11-008415	Phase 1/ongoing, not recruiting/April 2018	na	na	Anthony Windebank, Mayo Clinic/na
**7**. Autologous bone marrow-derived mesenchymal stem cells/na	Na/Intrathecal (via a standard lumbar puncture)	Every 2 months up to 1.5 year of the trial	Poland/University of Warmia and Mazury/NCT02881489; UWM/ALS-MSC.2015/002	Phase 1/Enrolling by invitation/April 2018	na	na	Wojciech Maksymowic/na
**8**. Autologous Bone Marrow Mononuclear Cells/ The average dose is 550 millions of cells (100–1,200 million) diluted in 2 ml saline. Placebo Comparator: Saline	Na/ Intramuscular infusion of autologous mononuclear **cells** (MNC) in TA muscle of one of the lower limb (randomly determined). Intramuscular infusion of 2 mL of saline (placebo) in the TA muscle of the contralateral limb (group control).	24 from baseline	Spain/Red de Terapia Celular/NCT02286011; TCIM/ELA2011-004801-25 (EudraCT Number)	Phase 1/ ongoing but not recruiting/December 2017	na	na	Joaquín A Gómez Espuch/na
**9**. Autologous bone marrow stem/progenitor cell/na	Na/intrathecally	1 year	Poland/Pomeranian Medical University Szczecin//NCT02193893; ZPO 02, ALS-BMSC #01 (Other Identifier: Department of General Pathology, PMU in Szczecin)	Phase 1/enrolling by invitation/December 2017	na	na	Boguslaw Machalinski, and Przemyslaw Nowacki/na
**10**. Adipose Derived Mesenchymal Stem Cell/2 × 10^6^ per kilogram	Na/intravenous	2 months	Iran, Islamic Republic/Royan institute/NCT02492516; Royan-Nerve-008	Phase 1/Completed/April 2017	na	na	Leila Arab/na
**11**. Autologous mesenchymal stem cells/10 × 106	Na/intraspinal injection	2 years	US/Mayo Clinic/NCT01142856; 09-001995	Phase/Completed/April 2011	na	na	Anthony J. Windebank,/na
**12**. Human Neural Progenitor Cells Secreting Glial Cell Line-Derived Neurotrophic Factor (CNS10-NPC-GDNF)/ two sequential dosing groups	NA/Stereotactic surgical device	12 months	US/Cedars-Sinai Medical Center and California Institute for Regenerative Medicine/ NCT02943850; Pro00042350	Phase 1/2a/recruiting/ April 2019	na	na	Robert H. Baloh/na
**13**. Bone marrow-derived mesenchymal stem/stromal cells/15 ± 4.5 x 10^6^/patient	Na/single intrathecal injection (via standard lumbar puncture)	3, 6, 9, 12, and 18 months	Czechia/Bioinova Ltd./EudraCT No. 2015-000139-33	Phase 1/2/Completed/ December 2015	safe procedure; reduction in ALSFRS decline at 3 months after application; 80% of the patients, FVC values remained stable or above 70% for a time period of 9 months.	30% of the patients experienced a mild to moderate headache, resembling the headaches after a standard lumbar puncture	Eva Sykova/(Sykova et al., 2017)
**14**. Human allogeneic Wharton's jelly-derived mesenchymal stem cells	Na/Intrathecal (via a standard lumbar puncture)	6 months - first time ALSFRS) + and then every 2 months up to 1.5 year of the trial	Poland/University of Warmia and Mazury/NCT02881476; UWM/ALS-MSC.2015/001	Phase 1/Enrolling by invitation/ December 2018	na	na	Wojciech Maksymowicz/na
**15**. Autologous bone marrow-derived stem cells/na		3 months intervals for 12 months	US/TCA Cellular Therapy/NCT01082653; 2008-ALS-I	Phase 1/suspended recruiting/ May 2014	na	na	TCA Cellular Therapy/na
**16**. Autologous mesenchymal stem cell/2 intrathecal autologous MSCs infusions (1 × 10^8^ cells)	Na/2 intrathecal autologous MSCs infusions	Intervalls 1, 3, 6, and 12 months	Brazil/ Hospital e Maternidade Dr. Christóvão da Gama/NCT02987413; HospitalMCG IEPSaoLucas	Phase 1/Completed/ August 2017	na	na	Leandro B Agati/na
**17**. Efficacy of Transplantation of Autologous Mesenchymal Stem Cells Secreting Neurotrophic Factors (MSC-NTF)/	Na/Combined intramuscular and intrathecal placebo administration	24 weeks post-transplantation Time Frame: Visits 1, 2, 3, 5, 6, 7, 8, 9, 10	US/Brainstorm-Cell Therapeutics/ NCT02017912; BCT-001-US	Phase 2/Completed/July 2016	na	na	Merit Cudkowicz/na
**18**. HLA-haplo matched Allogenic Bone Marrow Derived stem cells(“HYNRCS-Allo-ALS-02 inj”)/1.0 × 10^6^ cells/kg	Intrathecal	12 months	/Hanyang University Seoul Hospital/ NCT03214146; HYNR-CS-Allo-02	Phase 1/ recruiting/ November 2018	na	na	Seung Hyun Kim/na
**19**. Autologous peripheral blood mononuclear cells	Na/Subarachnoid Space	Week 1, week 2, week 4, week 12 after operation	China/The First Affiliated Hospital of Dalian Medical University/NCT03085706; DalianMU_003	Na/completed/March 2017	na	na	Jing Liu/na
**20**. Autologous CD4+ CD25+ regulatory T cells with concomitant subcutaneous IL-2 injections/Tregs (1 × 10^6^ /kg) with concomitant subcutaneous IL-2 injections (2 × 10^5^ IU/m^2^) 25 days (±2 days) post leukapheresis	Na/4 infusions of autologous expanded Tregs with concomitant subcutaneous injections of IL-2	3 months post-treatment for a total of two years	US/ The Methodist Hospital System/NCT03241784; Pro00013616	Phase 1/ongoing, not recruiting/ February 2018	na	na	Stanley H. Appel/na
**21**. Autologous bone marrow-derived stem cells(“HYNR-CS inj”)/na	Intrathecal	Week 12, −8, −4, 0, 4, 8, 12, 16	Korea Rep./Corestem, Inc./NCT01363401; HYNR_CS_ALS201	Phase 1/2/Completed/ February 2017	na	na	Seung Hyun Kim/na
**22**. Induced Pluripotent Stem Cells From an Existing Collection of Human Somatic Cells/na	Derivation for research purposes	na	Israel/ Hadassah Medical Organization/ NCT00801333; 0511-08-HMO	/ recruiting/December 2020	na	na	Benjamin Reubinoff/na
**23**. Autologous mesenchymal stem cells/na	2 intrathecal injections	10 months	Brazil/University of Sao Paulo General Hospital/NCT02917681;401922/2014-6	phase 1/2/recruiting/ February 2019	na	na	Gerson Chadi/na
**24**. Autologous Purified Bone-Marrow-Derived Stem Cell/na	Combined intravenous and intrathecal	4 months	Jordan/ Stem Cells Arabia/NCT03067857;SCA-MND1	Phase 1/2/Ongoing, not recruiting/ January 2019	na	na	Stem Cells Arabia/na
**25**. Autologous mesenchymal stem cells/administration of MSC: 1 million MSC/kg, 2 million MSC/kg and 4 million MSC/kg.	Intravenous administration of MSC/ placebo	6 months	Spain/Andalusian Initiative for Advanced Therapies - Fundación Pública Andaluza Progreso y Salud/ NCT02290886; CeTMAd/ELA/2011	Phase 1/ 2/recruiting/February 2021	na	na	Óscar Fernández/na
**26**. Autologous Bone Marrow Stem Cells/unknown	>6 months and <36 months/I.S. (T3-4) and intrathecal via laminectomy injection of cell fraction (placebo, saline solution)	Every 3 months	Spain/Fundacion para la Formacion e Investigacion Sanitarias de la Region de Murcia /NCT01254539; Extension CMN/ELA 2006-003096-12 (EudraCT Number) EC07/90762 (Other Identifier: ISCIII)	Phase 1/2/Completed/November 2015	na	na	Jose María Moraleda Jiménez/na
**27**. Mononuclear autologous bone marrow cells/unknown	After 6 months/2x I.S. ipsi/contralateral (posterior funiculus) after laminectomy	Every 3 months 1 year	Spain/ Fundacion para la Formacion e Investigacion Sanitarias de la Region de Murcia /NCT00855400	Phase 1 and 2/ completed/2010	Safe. Greater number of MNs in treated segments (neurotrophic effect). MNs surrounded with CD90+ cells and did not show degenerative ubiquitin deposits.	No severe transplant-related adverse event. Adverse events grade ≤ 2.	Jose Maria Moraleda Jiménez (Blanquer et al., 2012)
**28**. Umbilical Cord Mesenchymal Stem Cells/?	>6 months and <36 months/4x (every 3–5 days) intrathecally	1, 6, 12, and 24months	China/General Hospital of Chinese Armed Police Forces/NCT01494480/20111207ALS	Phase 2/recruiting by invitations/2015	na	na	Dr. Yi Hua An/na
**29**. Human Spinal Cord Derived Neural Stem Cell/Group A: 3 ambulatory early-stage subjects with arm weakness but not paralysis, to receive bilateral C3 through C4 injections of 2 × 10^6^ cells (10 injections × 2x10^5^ cells/ injection)	<24 months/I.S.	2 and 4 weeks, and then at 3, 6, 9, 12, 15, 18, 21, and 24 months, and then at every 6 months thereafter until death.	US/Neuralstem Inc./NCT01730716; NS2012-3	Phase 2/unknown/April 2012	na	na	Neuralstem Inc/na
**Group B:** 3 ambulatory early-stage subjects with arm weakness but not paralysis, to receive bilateral C3 through C5 injections of 4 × 10^6^ cells (20 injections × 2 × 10^5^ cells/injection) **Group C:** 3 ambulatory early-stage subjects with arm weakness but not paralysis, to receive bilateral C3 through C5 injections of 6 × 10^6^ cells (20 injections × 3 × 10^5^ cells/injection) **Group D:** 3 ambulatory early-stage subjects with arm weakness but not paralysis, to receive bilateral C3 through C5 injections of 8 × 10^6^ cells (20 injections × 4 × 10^5^ cells/ injection) **Group E:** 3 ambulatory early-stage subjects with arm weakness but not paralysis, to receive bilateral L2 through L5 injections of 8x10^6^ cells (20 injections of 4 × 10^5^ cells/ injection) and then ~4–12 weeks later to receive bilateral C3 through C5 injections of 8 × 10^6^ cells (20 injections of 4 × 10^5^ cells/ injection.							
**30**. Human Spinal Cord Derived Neural Stem Cell/5 injection × 100,000 neural cells	<24 months/unilateral and bilateral intraspinal lumbar microinjection	1, 3, 6, 9, 12, 18, 24, 30, 36, 42, and 48 month follow-up/post-surgery visits	US/Neuralstem Inc./NCT01348451; NS2008-1	Phase 1/ongoing, not recruiting by invitations/March 2016	Twelve patients have received a transplant. By discharge, none had a documented motor function decrement. The procedural safety of unilateral and bilateral intraspinal lumbar microinjection was proved. Completion of phase I safety trial is planned by proceeding to cervical and combined cervical + lumbar microinjections in ALS patients	One instance of transient intraoperative somatosensory-evoked potentials depression. In the immediate postoperative period-−1 episode of urinary retention requiring Foley catheter reinsertion. 2 patients required readmission and reoperation for cerebrospinal fluid leak or suprafascial wound dehiscence (*n* = 1 each). Two deaths occurred at 8 and 13 months postsurgery; neither was related to the surgical transplant.	Neuralstem Inc./(Glass et al., 2012; Riley et al., 2012)
**31**. Human fetal neural stem cells/	<6months/I.S. microinjection	36 months	Italy/Azienda Ospedaliera Santa Maria, Terni and Università di Padova Italy /NCT01640067	Phase 1/completed/December 2015	Safe procedure; no increase of disease progression up to18 months; transitory improvement of the subscore ambulation on the ALS-FRS-R scale (from 1 to 2) in 2 patients; improvement of the MRC score for tibialis anterior in 1 patient (for 7 months); the latter and 2 additional patients refused PEG and invasive ventilation and died 8 months after surgery due to the progression of respiratory failure (confirmed by autopsies)	na	Angelo L Vescovi/(Gelati et al., 2013; Mazzini et al., 2015)
**32**. HLA-haplo Matched Allogenic Bone Marrow Derived Stem Cell(“HYNR-CS-Allo Inj”)/0.25/0.5/1 × 10^6^ cells/kg dose cohort	<5 years/2x Intrathecal injection with 28 days interval	4, 8, 12, 16 weeks	Korea/ Hanyang University Seoul Hospital; Corestem, Inc./NCT01758510; HYNR-CS-Allo-01	Phase 1/ ongoing/December 2017	na	na	Seung Hyun Kim/na
**33**. Bone Marrow Derived Mesenchymal Stem Cell/	<2 years/Intraventricular	1, 3, 6, and 12 months	Islamic Republic of Iran/Royan Institute/ NCT01759784	Phase 1/Withdrawn prior enrolment/December 2015	Withdrawn prior enrolment	na	Chair: Hamid Gourabi; Director: Nasser Aghdami/na
**34**. Bone Marrow Derived Mesenchymal Stem Cell/	<2 years/Intravenous	1, 3, 6, and 12 months	Islamic Republic of Iran/Royan Institute/NCT01759797; Royan-Nerve-005	Phase 1/Completed/January 2014	na	na	Chair: Hamid Gourabi Director: Nasser Aghdami, Director: Seyed Masoud Nabavi/na
**35**. Bone Marrow Derived Mesenchymal Stem Cell/na	<2 years/Intrathecal	1, 3, 6 and 12 months	Islamic Republic of Iran/Royan Institute/NCT01771640; Royan-Nerve-006	Phase 1/Completed/December 2015	na	na	Hamid Gourabi/na
**36**. Autologous cultured mesenchymal bone marrow stromal cells secreting neurotrophic factors (MSC-NTF)/ 94 × 10^6^, 141 × 10^6^, and 188 × 10^6^ cells (depending on the groups)	<2 years/single Intrathecal plus multiple (24 sites) intramuscular	Monthly, than every 6 months	Israel/Hadassah Medical Organization; Brainstorm-Cell Therapeutics/NCT01777646; MSC-NTF-002-HMO-CTIL	Phase 2a/recruiting/2014	The treatment was found to be safe and well-tolerated over the study follow-up period; Of 14 patients, 13 (87%) were defined as responders to either ALS FRS-revised or forced vital capacity, having at least 25% improvement at 6 months after treatment in the slope of progression.	Most of the adverse effects were mild and transient	Dimitrios Karusis/(Petrou et al., 2016)
**37**. Autologous cultured mesenchymal bone marrow stromal cells secreting neurotrophic factors (MSC-NTF)/Intramuscular 24 × 10^6^; Intrathecal 60 × 10^6^	<2 years/single Intrathecal; intramuscular (24 sites)	Monthly, than every 6 months, 12 months	Israel/Hadassah Medical Organization/NCT01051882; MSC-NTF-001-HMO-CTIL	Phase 1 and 2/completed/January 2014	na	na	Dimitrios Karussis/(Petrou et al., 2016)

Unfortunately, majority of the granted trials did not perform patients' follow-up longer than 24 months. The first long-term follow-up after nearly 9 years of patients monitoring, showed that dorsal application of MSC is safe procedure with no clear clinical benefits, but also without any structural changes or deterioration in psychosocial status (Mazzini et al., 2003, 2010, 2011). Trial with the mononuclear CD133(+) fraction isolated from the peripheral blood after the frontal motor cortex grafting were reported to significantly extend life of ALS patients and to improve their lifestyle compared with patients that were not treated with the cells (Martinez et al., 2009). Deda et al. reported that one year after the implantation of hematopoietic progenitor stem cells significantly improved tested parameters in 70 percent of the treated patients, compared with their pre-operative status (Deda et al., 2009). A latest prospective, non-randomized, open label clinical trial has been completed in 2016 in Prague, Czech Republic (Sykova et al., 2017). Study evaluated the safety and the efficacy of autologous multipotent MSC in the patients with confirmed diagnosis of ALS (http://www.sukl.eu). The trial involved 26 patients with sporadic ALS, who received a single intrathecal (via a lumbar puncture) dose of autologous MSCs applied via a lumbar puncture into the cerebrospinal fluid. As compared to previous trials, this study included the largest group of ALS patients, had a longer pre-and post-treatment assessment period, and a relatively small dose of stem cells was used. A potential adverse reactions were assessed by clinical, laboratory and MR examination for 18 months. Patients underwent clinical evaluation using ALS functional rating scale (ALSFRS), Norris spinal and bulbar scale (NSS and NSB), forced vital capacity (FVC) and weakness scale (WS). This study showed that 30 percent of the patients experienced mild/moderate headache after MSCs application, not connected with the actual cell application. No suspected serious adverse reactions or new cerebrospinal pathology on MR examinations were observed. Eighty percent of patients preserved FVC values above 60 percent for 12 months. Fourteen patients (out of 26) with a remarkable pretreatment decline in functional scales, had significant reduction/stabilization in their total functional score decline at 3 months after application, which was less pronounced at 6 and 9 months.

Despite an increasing number of clinical trials proving the safety of the procedure there is a great need for bigger multicentre trials. Even though some small series of experiments involving patients showed an improvement of motor and sensory functions after the administration of stem cells, there is a need for bigger multicentre studies with placebo group of patients. The above trials that resulted with a neuroprotective effect after cell-based therapy have employed various routes of application, different type of cells, and not same ways of clinical evaluation, therefore there is a need for unification of future clinical trials design. One could also speculate that the combination of different routes of cell delivery might bring even better results related to survival and motor functions. We would like to stress once again, that current research should also employ physiological characteristics of all cells types that are supposed to be delivered into patients. This could give us more homogenous data between the trials as well as develop an algorithm that will enable prognosis of cell-based therapy in the future (Forostyak et al., 2016b). Finally, specific markers, which will enable early disease diagnosis, are of a great importance for the successful cell-based therapy, mainly because at the beginning of neurodegeneration stem cells might bring more benefits in rescuing neurones from inevitable death, if compared with the therapy at the terminal-stage of ALS.

## Author contributions

SF and ES - data collection and literature overview, manuscript writing.

### Conflict of interest statement

The authors declare that the research was conducted in the absence of any commercial or financial relationships that could be construed as a potential conflict of interest.
